# Severe Sepsis Associated with Lemierre's Syndrome: A Rare but Life-Threatening Disease

**DOI:** 10.1155/2016/1264283

**Published:** 2016-04-05

**Authors:** Audrey Tawa, Raphaëlle Larmet, Yannick Malledant, Philippe Seguin

**Affiliations:** ^1^Intensive Care Unit, Anaesthesia and Critical Care Department, University Hospital of Rennes, 2 rue Henri Le Guilloux, 35000 Rennes, France; ^2^Inserm U991, Rennes, France

## Abstract

*Background.* The incidence of Lemierre's syndrome has increased in the past decade. This posttonsillitis complication may be life threatening.* Case Presentation.* A 19-year-old patient was admitted to Surgical Intensive Care Unit of a French University Hospital for high fever, low blood pressure, and haemoptysis following a sore throat episode. Blood analysis revealed a thrombopenia, an acute renal failure, and an elevated lactate serum.* Fusobacterium necrophorum *was found in blood cultures. Computed tomography of the neck and lungs confirmed the diagnosis of Lemierre's syndrome: pleural effusions, bilateral lung infiltrates, and an internal jugular vein thrombosis. Fluid administration and antibiotic treatment were quickly initiated. Patient left the unit four days after his admission without any organ dysfunction.* Conclusion.* Lemierre's syndrome may lead to multiorgan dysfunction and should be rapidly identified.

## 1. Introduction

Lemierre's syndrome is an uncommon disease related to anaerobia septicaemia. The primary oropharyngeal infection (ear, nose, or throat) extends to the internal jugular vein and spreads in distal septic emboli to other organs and particularly to the lungs (80 to 100%) one to three weeks later.

## 2. Case Presentation

A previously healthy, 19-year-old Caucasian man reported to his attending physician a recent odynophagia, left cervical pain, and fever. At this time, he was diagnosed with a nonbacterial pharyngitis (a rapid strep test was negative) and treated with acetaminophen.

**Figure 1:**
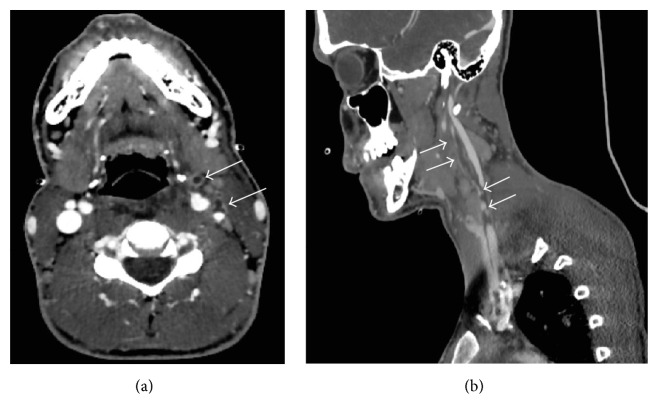
Left jugular vein thrombosis associated with thrombosis of collateral veins (white arrows in panels (a) and (b)) and cervicofacial cellulitis.

**Figure 2:**
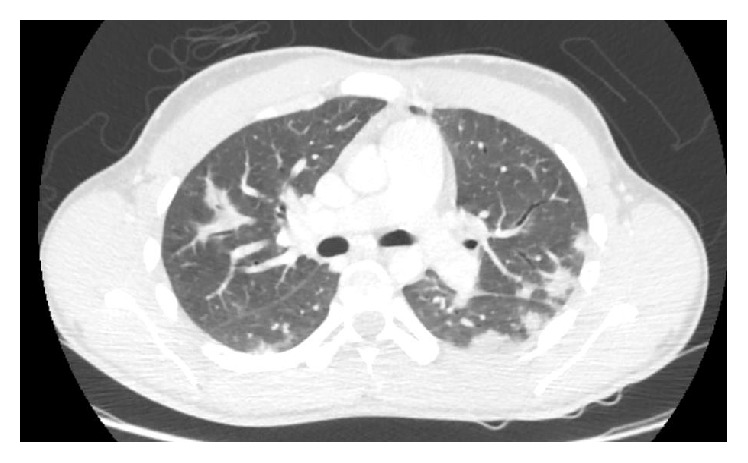
Multiple pulmonary nodules.

Four days later, he was admitted to the Surgical Intensive Care Unit of a French University Hospital because of severe sepsis with persistent fever and asthenia. Physical examination revealed a high fever (39°6), a low blood pressure (78/40 mmHg) with diffuse mottles, and respiratory failure (SpO_2_ 88% while breathing room air) associated with haemoptysis. The ear, nose, and throat examination was normal and a tender induration was found on the left and upper side of the neck. Laboratory test revealed a thrombopenia (14 G/L), a high white blood cell count (14.1 G/L), a moderate acute renal injury (creatinine of 97 *µ*mol/L and urea of 11.5 mmol/L), and an elevated lactate serum of 1.8 mmol/L (normal value < 1.2 mmol/L). A chest and neck computed tomography (Figures 1 and 2) revealed a retropharyngeal abscess, an internal jugular vein thrombosis, bilateral lung infiltrates, and pleural effusions. Ceftriaxone associated with metronidazole and gentamicin was introduced right after blood cultures were drawn. Few hours later, severe sepsis regressed after consequent fluid administration and platelet transfusion. Only medical treatment was necessary since the abscess was too profound to reach.* Fusobacterium necrophorum *and* Streptococcus constellatus *were found in blood cultures. Initial antibiotic therapy was replaced by high doses of amoxicillin (8 g), clavulanate, and metronidazole. The patient left the unit for the Infectious Diseases Unit four days after admission. Anticoagulant treatment by heparin was initiated on the fifth day when thrombopenia was corrected. Oral anticoagulant was introduced secondarily. Apyrexia was obtained on the seventeenth day. Antibiotic therapy was maintained for three weeks.

One month later, pulmonary nodules had mostly regressed on the lung computed tomography. Recanalization of the internal jugular vein happened only ten weeks after anticoagulant treatment was introduced.

## 3. Discussion

Here we describe an unusual severe presentation of Lemierre's syndrome with respiratory failure, low blood pressure, thrombopenia, and acute renal injury.

In 1936, a French doctor named Lemierre who worked in the Claude Bernard Hospital in Paris described a posttonsillitis septicaemia among 20 patients [1]. He isolated one of the causal anaerobia pathogens* Fusobacterium necrophorum* in necrotizing tissue. Fifty years later, a case report of a posttonsillitis septicaemia was named after him [1].

Lemierre's syndrome is mainly reported in young patients. Its incidence is estimated at 14.4 cases/million/year in the 15–24-year-olds whereas it reaches 1.4 cases/million/year in people over 40 years old [2]. This incidence has increased in these past decades related to a more restrictive antibiotic therapy policy for oropharyngeal infection [3, 4]. Its mortality rate while treated is high (5 to 18%) especially since it affects young and healthy patients [5, 6]. Two cases of sexually transmitted Lemierre's syndrome have been described in older patients [7, 8].

The physiopathology is not well established and few hypotheses are reported [5]. First of all, the bacteria might spread through the hematogenous pathway to the tonsillar vein and then to the systemic circulation [3]. Another hypothesis suggests that infection might extend to the pharyngeal space through lymphatic system [9]. Finally, a primary bacterial or viral infection (especially* Epstein-Barr Virus*) may injure pharyngeal mucosa permitting* Fusobacterium necrophorum *to extend to the intern jugular vein [9, 10]. This last theory could partially explain the higher incidence among young adults.

The diagnosis stands on a primary oropharyngeal infection, evidence of cervical venous thrombosis, and at least one distant septic embolus. Blood (or a sterile site) cultures are commonly positive to* Fusobacterium necrophorum *but* Streptococcus oralis, *groups B and Cstreptococci,* Staphylococcus epidermidis, Enterococcus *sp.,* Proteus mirabilis*, and/or* Bacteroides *sp. may also be found [2]. In 80% of cases, the causal agent grows in blood cultures but could require a long growth time [3, 11]. The optimal antimicrobial therapy associates a *β*-lactam and metronidazole during 3–6 weeks. Antimicrobial resistance is unusual but few strains of* F. necrophorum *with *β*-lactamase production have been reported [12]. Oral streptococci should also be targeted [5]. Persistent fever is common even with appropriate treatment because antibiotics have trouble penetrating the clot [5].

Septic or sterile arthritis (13 to 27%), skin and soft tissue infections (5 to 16%), and osteomyelitis (less than 9%) are less usual presentations [5]. Cerebral abscesses and meningitis may also occur by retrograde extension [13]. Vertebral thrombosis with spondylitis and epiduritis have also been described [11]. Hemagglutinin production induces platelet aggregation leading to diffuse intravascular coagulation and thrombocytopenia [4].

The tonsil examination may be normal and local signs subtle when metastatic complications occur [14]. Patient's general condition may be altered because of severe sepsis. One case of isolated low blood pressure without any other organ dysfunction has been reported [6]. One septic shock associated with Lemierre's syndrome has been described, and the causal pathogen was not usual anaerobia bacteria but* Staphylococcus aureus* [15].

Doppler ultrasonography is very specific to identification of the thrombosis. However, its sensitivity is low in thrombus below the mandible, thrombus under the clavicle, and newly formed thrombus because of its low echogenicity. Computed tomography of neck and chest with contrast remains the best tool to diagnose retropharyngeal abscess, internal jugular vein thrombosis, and septic pulmonary emboli [5]. Moreover, it allows diagnosing and precising distant lesions.

Surgical debridement and drainage could be useful when abscesses are identified on the computed tomography. Surgical ligation of the internal jugular vein might be indicated in case of uncontrolled septic emboli despite adequate antibiotic treatment and anticoagulation [5]. However, anticoagulation therapy remains controversial. Indeed, the risks and benefits of anticoagulation therapy cannot be assessed in randomized, controlled trial because of Lemierre's syndrome low incidence [4]. Some authors suggest that anticoagulant treatment should only be introduced when a retrograde sinus cavernous thrombosis exists in order to prevent cerebral complications [16]. Other physicians recommend its use in context of extensive thrombosis. Recanalization of the internal jugular vein in patients only treated with antibiotics is uncommon but has been reported [17].

To conclude, we reported a multiorgan dysfunction related to Lemierre's syndrome. Its incidence seems to be increasing because of a more restrictive antibiotic therapy prescription. Practicians should quickly identify this life-threatening disease in the emergency room.

## References

[1] Lemierre A. (1936). On certain septicaemias due to anaerobic organisms. *The Lancet*.

[2] Hagelskjær Kristensen L., Prag J. (2008). Localised *Fusobacterium necrophorum* infections: a prospective laboratory-based Danish study. *European Journal of Clinical Microbiology & Infectious Diseases*.

[3] Riordan T. (2007). Human infection with *Fusobacterium necrophorum* (Necrobacillosis), with a focus on Lemierre's syndrome. *Clinical Microbiology Reviews*.

[4] Karkos P. D., Asrani S., Karkos C. D. (2009). Lemierre's syndrome: a systematic review. *Laryngoscope*.

[5] Kuppalli K., Livorsi D., Talati N. J., Osborn M. (2012). Lemierre's syndrome due to *Fusobacterium necrophorum*. *The Lancet Infectious Diseases*.

[6] Prakashchandra S. P., Patel A. K. B., Patel K., Raj Kumar Doshi P., Patel N. A. (2015). Grave complication of pharyngitis: Lemierre syndrome. *Journal of Clinical and Diagnostic Research*.

[7] Takenouchi S., Kunieda T., Yamada R., Yamakita N. (2014). Lemierre syndrome caused by oral sex. *Journal of the Formosan Medical Association*.

[8] Dellamonica P., Bernard E. (1999). ‘Deep throat’ cellulitis. *Journal of Infection*.

[9] Hughes C. E., Spear R. K., Shinabarger C. E., Tuna I. C. (1994). Septic pulmonary emboli complicating mastoiditis: Lemierre's syndrome revisited. *Clinical Infectious Diseases*.

[10] Armstrong A. W., Spooner K., Sanders J. W. (2000). Lemierre's syndrome. *Current Infectious Disease Reports*.

[11] Medina F., Tatay M., Smati M. (2015). Lemierre's syndrome: an unusual presentation. *Medecine et Maladies Infectieuses*.

[12] Brook I., Wexler H. M., Goldstein E. J. C. (2013). Antianaerobic antimicrobials: spectrum and susceptibility testing. *Clinical Microbiology Reviews*.

[13] Sibai K., Sarasin F. (2004). Lemierre syndrome: a diagnosis to keep in mind. *Revue Medicale de la Suisse Romande*.

[14] Lustig L. R., Cusick B. C., Cheung S. W., Lee K. C. (1995). Lemierre's syndrome: two cases of postanginal sepsis. *Otolaryngology—Head and Neck Surgery*.

[15] Marulasiddappa V., Tejesh C. A. (2013). Lemierre′s syndrome presenting with septic shock. *Indian Journal of Critical Care Medicine*.

[16] Bondy P., Grant T. (2008). Lemierre's syndrome: what are the roles for anticoagulation and long-term antibiotic therapy?. *Annals of Otology, Rhinology and Laryngology*.

[17] Agrafiotis M., Moulara E., Chloros D., Tsara V. (2015). Lemierre syndrome and the role of modern antibiotics and therapeutic anticoagulation in its treatment. *American Journal of Emergency Medicine*.

